# Intranasal administration of exosomes derived from mesenchymal stem cells ameliorates autistic-like behaviors of BTBR mice

**DOI:** 10.1186/s13229-018-0240-6

**Published:** 2018-11-21

**Authors:** Nisim Perets, Stav Hertz, Michael London, Daniel Offen

**Affiliations:** 1 Sagol School of Neuroscience, Tel Aviv University, Tel Aviv, Israel; 2 Edmond and Lily Safra Center for Brain Sciences, Hebrew University, Jerusalem, Israel; 3 Sacklar School of Medicine, Department of Human Genetics and Biochemistry, Tel Aviv University, Tel Aviv, Israel

## Abstract

Autism spectrum disorders (ASD) are neurodevelopmental disorders characterized by three core symptoms that include social interaction deficits, cognitive inflexibility, and communication disorders. They have been steadily increasing in children over the past several years, with no effective treatment. BTBR T+tf/J (BTBR) mice are an accepted model of evaluating autistic-like behaviors as they present all core symptoms of ASD. We have previously shown that transplantation of human bone marrow mesenchymal stem cells (MSC) to the lateral ventricles of BTBR mice results in long lasting improvement in their autistic behavioral phenotypes. Recent studies point exosomes as the main mediators of the therapeutic effect of MSC. Here, we tested whether treatment with the exosomes secreted from MSC (MSC-exo) will show similar beneficial effects. We found that intranasal administration of MSC-exo increased male to male social interaction and reduced repetitive behaviors. Moreover, the treatment led to increases of male to female ultrasonic vocalizations and significant improvement in maternal behaviors of pup retrieval. No negative symptoms were detected following MSC-exo intranasal treatments in BTBR or healthy C57BL mice. The marked beneficial effects of the exosomes in BTBR mice may translate to a novel, non-invasive, and therapeutic strategy to reduce the symptoms of ASD.

**Electronic supplementary material:**

The online version of this article (10.1186/s13229-018-0240-6) contains supplementary material, which is available to authorized users.

## Introduction

Autism spectrum disorders (ASD) are neurodevelopmental disabilities characterized by three core symptoms: severe impairment of social interactions and communication skills, increased repetitive behaviors, and cognitive inflexibility [1]. In this study, we used the inbred mouse strain BTBR T+tf/J (BTBR) that incorporates multiple behavioral phenotypes relevant to all three diagnostic symptoms of autism. BTBR present significantly reduced social approach, low reciprocal social interactions, and impaired juvenile play in comparison to the C57BL controls [2, 3]. Using this model, we have recently shown that MSC transplantation to the lateral ventricles of the brain of BTBR mice has the ability to ameliorate their core autistic-like behaviors [4]. Moreover, the behavioral effect of MSC on BTBR mice had lasted for 6 months despite the fact that the MSC themselves do not survive in the transplanted tissue for such an extended period [5]. Such phenomenon has been reported in the literature under the title “hit and run” meaning MSC have the ability to leave a lasting “memory” on the tissue, even after the MSC cells have been degraded [6]. It has been suggested that the MSC therapeutic effect is mainly mediated by their secretome to the tissue [7]. The secretome is referred to as the complete repertoire of molecules and extracellular vesicles secreted from MSC. Yet, it has been found that the nano-vesicles, exosomes, are the major mediators between the MSC and the tissue [8, 9].

Exosomes were initially thought to be a mechanism for removing unneeded membrane proteins from reticulocytes. However, recent studies have shown that they are also used for cell to cell communication through the carrying of genetic information from between cells [10]. Several studies have reported that MSC-exo have functions similar to those of MSC, such as repairing damaged tissue, suppressing inflammatory responses, and modulating the immune system [11, 12]. Exosomes contain mainly proteins, as well as RNA and a large number of micro RNAs. Approximately, 25% of these proteins and RNA play a role in cell growth and maintenance [13, 14]. A key advantage of MSC-exo over MSC is their ability to enter the brain with ease following intranasal administration [15, 16]. We have previously demonstrated that exosomes loaded with gold nanoparticles can cross the blood-brain barrier via intranasal administration and can be visualize in vivo inside the brain [17].

Here, we show that BTBR mice that were treated with MSC-exo via intranasal administration present significant improvement in the social interaction domain, ultrasonic communication, and repetitive behavior. Moreover, we show for the first time that BTBR mothers that were treated with MSC-exo presented significant improvement in maternal pup retrieval behavior.

## Results

### MSC-exo characterization

**Fig. 1:**
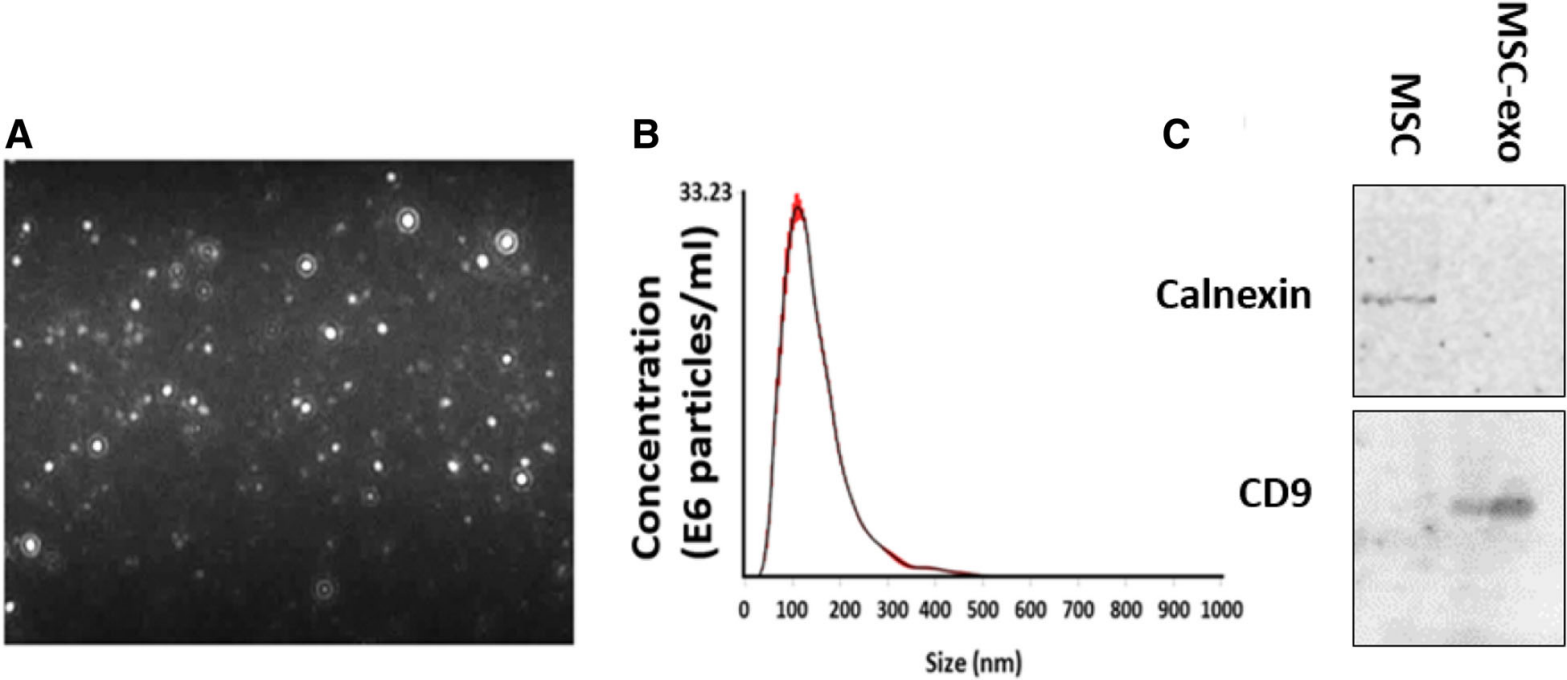
Characterization of MSC-exo. **a** Visualization of the exosomes using NanoSight technology. **b** Analysis of size distribution and concentrations. **c** Western blot analysis of CD9 which exists in exosomes but not in the MSC lysate in contrast to Calnexin which is absent in exosomes and found in the MSC lysate

MSC-exo were characterized by NanoSight. The average size was 114 ± 2.9 nm, and the average concentration was 3.81 × 10^8^ particles/μL (Fig. 1a, b). Western blot analysis indicates that the MSC-exo express CD9 and CD63, while we could not detect it in the MSC lysate (not shown). In contrast, Calnexin was undetectable in the MSC-exo and found in the MSC lysate, indicating for the purity of the exosomes [18] (Fig. 1c).

### MSC-exo improves male to male social interaction and reduces repetitive behaviors during social interaction

**Fig. 2:**
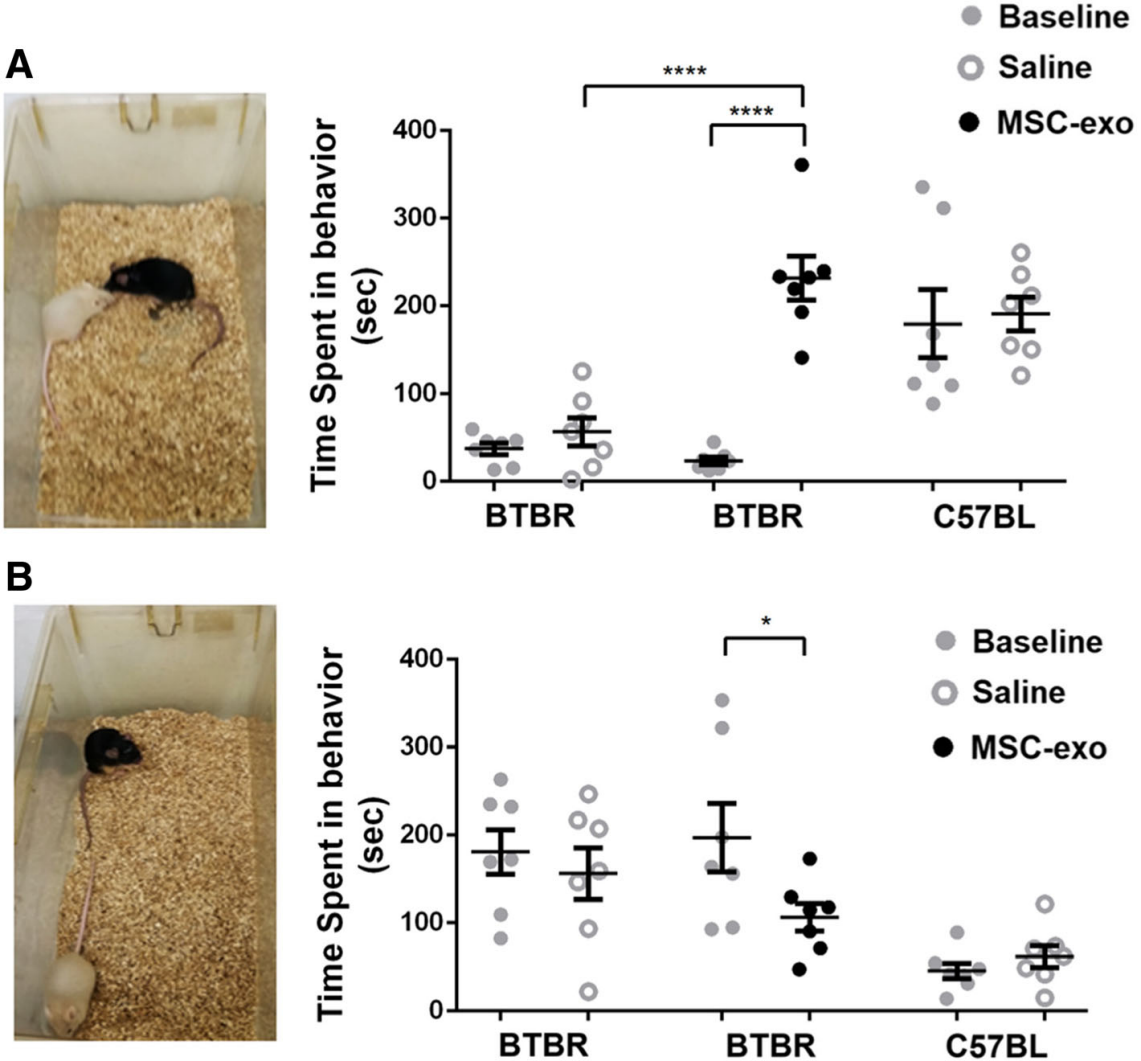
MSC-exo increased male to male social interaction and repetitive behaviors during social interaction. Each group was tested for basal behaviors (gray) and was re-tested 3 weeks after treatment (saline or MSC-exo, black). **a** Intra-subject comparison showed that the BTBR MSC-exo group had spent a significantly longer time engaging in social interaction with other stranger male mice (paired T-test). Inter-group comparison showed that the BTBR MSC-exo group had spent significantly more time engaging in social interaction compared to BTBR saline (ANOVA1, Bonferroni). **b** Intra-subject comparison of BTBR MSC-exo group had spent significantly less time in repetitive behaviors (paired *T* test). Inter-group comparison showed BTBR MSC-exo is not significantly different than both BTBR saline and C57BL saline groups in time spent in repetitive behaviors. (ANOVA1, Bonferroni). The data is presented as mean + SEM. ***p < 0.001

Mice were tested for male to male interaction before and after MSC-exo or saline administration. Intra-subject analysis showed that the BTBR MSC-exo mice group had spent significantly more time in social interactions after the treatment whereas the BTBR saline and C57BL saline groups did not show any change (paired *t* test, *t*_5_ < 0.0001). Comparison analysis between the groups showed that the BTBR mice of both groups had spent significantly less time engaging in social interaction in comparison to C57BL basal behavior (ANOVA1, *F*_2,18_ = 14.4, *p* < 0.01, Bonferroni). Exosome treatment dramatically increased social interactions in the BTBR MSC-exo group in comparison to BTBR mice treated with saline and is comparable to the C57BL mice group (ANOVA1, *F*_2,16_ = 9.44, *p* < 0.01, Bonferroni) (Fig. 2a, Additional files 1 and 2).

During the social interaction test, the repetitive behavior was also measured. Intra-subject analysis showed that the BTBR MSC-exo-treated group spent significantly less time in repetitive behaviors while both the BTBR saline and C57BL saline mice groups did not show any changes in comparison to their basal behaviors (paired *t* test, *t*_5_ < 0.001). A comparison analysis between groups showed that before treatment, there was no difference between BTBR MSC-exo and BTBR saline basal behaviors, and that both groups significantly differed from the basal behavior of the C57BL saline group (ANOVA1, *F*_2,18_ = 13.71, *p* < 0.001, Bonferroni). After treatment, The BTBR MSC-exo group was not significantly different from the BTBR saline group and the C57BL group (Fig. 2b). In repetitive behavior tests that were outside of the context of social interaction, BTBR MSC-exo spent significantly less time in repetitive grooming and digging in intra-subject analysis compared to their basal behaviors (*t*_5_ < 0.05). In groups, comparisons showed that they were significantly different from the BTBR saline group but not from the C57BL saline group (ANOVA1, *F*_2,18_ = 13.83, *p* < 0.01, Bonferroni) (Additional file 5: Figure S1). Importantly, C57BL MSC-exo mice did not present any behavioral differences in neither social interactions, antisocial interactions, nor repetitive behaviors during social contact (Additional file 5: Figure S2A). In comparison between MSC-exo to exosomes isolated from neuronal stem cells (NSC-exo), we found that only the MSC-exo-treated group presented a significant increase in social interaction, a reduction in repetitive behaviors, and a significant increase in communication while NSC-exo-treated mice did not present the same behavioral differences compared to saline-treated group (ANOVA1, *F*_2,14_ = 4.28, *p* < 0.05, Bonferroni). Importantly, MSC-exo and saline groups were also used as a biological replication of the social interaction with different seven mice per group (Additional file 5: Figure S3).

### MSC-exo improved male to female ultrasonic vocalizations

**Fig. 3:**
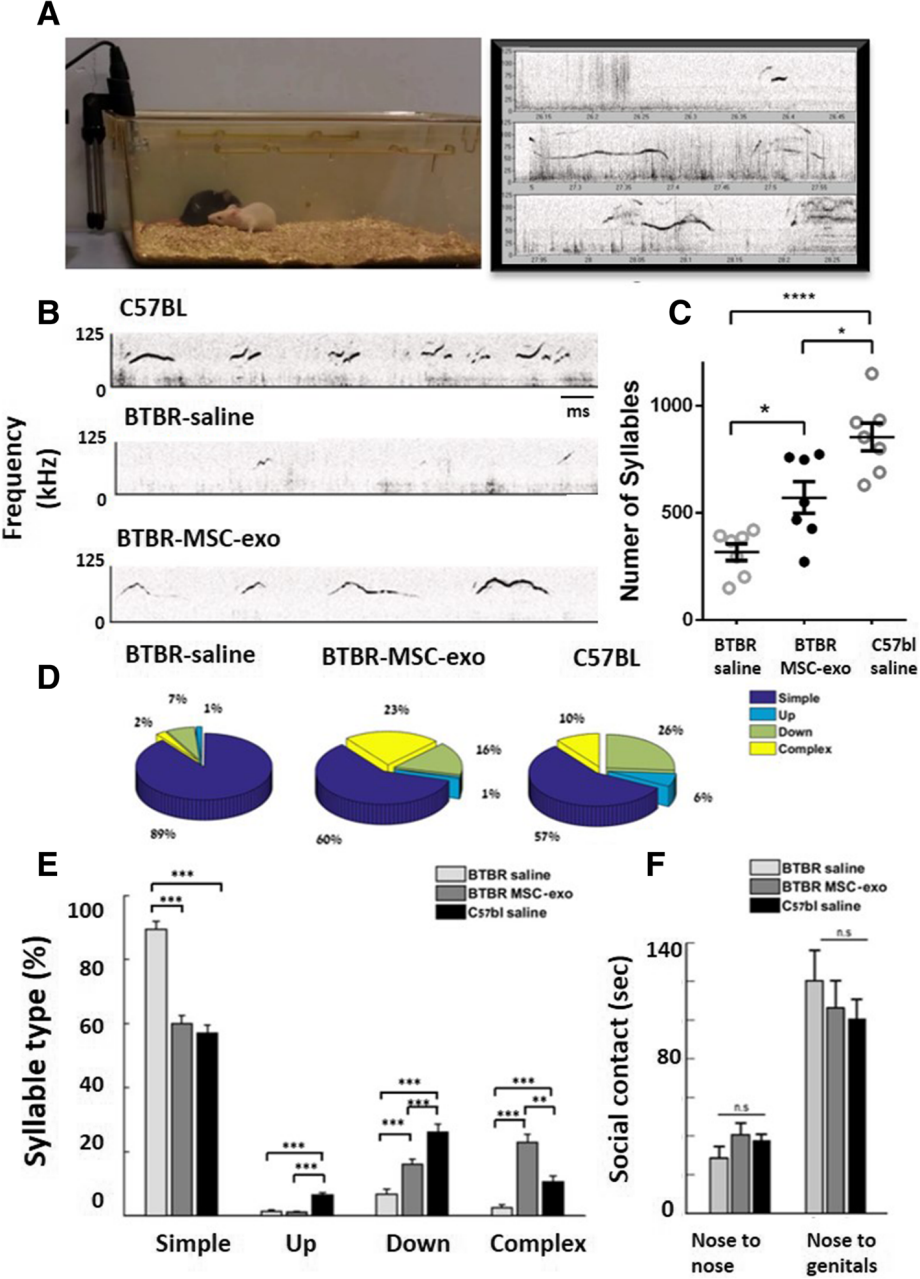
Specific improvement of ultrasonic vocalizations after intranasal administration of MSC-exo. **a** visualization of the experimental set, right panel: male and female mice in courtship meeting. Left panel: real-time spectrogram of ultrasonic vocalizations. **b** Example of differences in spectrogram of C57BL, saline-treated, and MSC-exo-treated mice suggests that MSC-exo-treated mice improved in complexity of syllables. **c** BTBR MSC-exo had more syllables of ultrasonic vocalizations compared to saline BTBR, yet less syllables than C57BL mice. **d** and **e** Automatic classification of syllables showed significant reduction in the use of simple syllables by BTBR MSC-exo and C57BL saline compared to BTBR saline. Classification also showed increased use in complex and down syllables by BTBR MSC-exo and C57BL saline compared to BTBR saline. **f** There was not a significant difference between the groups in social contact (nose to nose and nose to genitals), indicating a specific effect on ultrasonic vocalizations. (ANOVA1, Bonferroni). Data is presented as mean + SEM. **p < 0.01, ***p < 0.001

In general, male mice emitted a large number of complex ultrasonic vocalizations (USVs) when interacting with adult females (Fig. 3a, experimental demonstration). As seen qualitatively from the spectrograms in our study, BTBR MSC-exo vocalizations became more complex and longer compared to the BTBR saline group, making them more similar to C57BL (Fig. 3b). During the first 5 min of interaction with females, BTBR saline mice emitted 317 ± 39.4 syllables, BTBR MSC-exo emitted 571 ± 74 (180% more), and C57BL emitted 854.5 ± 65.2 syllables (Fig. 3c, ANOVA1, *F*_2,18_ = 19.2, *p* < 0.001, Bonferroni). Classification of syllables revealed significant differences between the syllable types used by BTBR saline and BTBR MSC-exo (Fig. 3d, e). For simple syllables, BTBR saline used 89%, BTBR MSC-exo used 57%, and C57BL saline used 60% (ANOVA1, *F*_2,18_ = 49.44, *p* < 0.001, Bonferroni). For complex syllables, BTBR saline used 2%, BTBR MSC-exo used 23%, and C57BL saline used 10% (ANOVA1, *F*_2,18_ = 30.61, *p* < 0.001, Bonferroni). For down syllables, BTBR saline used 7%, BTBR MSC-exo used 16%, and C57BL saline used 26% (ANOVA1, *F*_2,18_ = 25.23, *p* < 0.001, Bonferroni). For up syllables, BTBR saline used 1%, BTBR MSC-exo used 1%, and C57BL saline used 6% (ANOVA1, *F*_2,18_ = 41.22, *p* < 0.001, Bonferroni).

There was no significant difference between groups in time spent sniffing the female’s genitals or faces, meaning that the effect seen in the ultrasonic vocalizations was not impacted by the pheromones of the females (Fig. 3f). C57BL mice that were treated with MSC-exo showed similar number of syllables compared to C57BL saline group (Additional file 5: Figure S2B). In comparison between MSC-exo and NSC-exo, only the BTBR MSC-exo group presented a significant increase in number of syllables compared to BTBR saline group (ANOVA1, *F*_2,14_ = 9.44, *p* < 0.01, Additional file 5: Figure S3B). This result also used as biological replication of the vocalizations test.

### MSC-EXO improves pup retrieval behavior

**Fig. 4:**
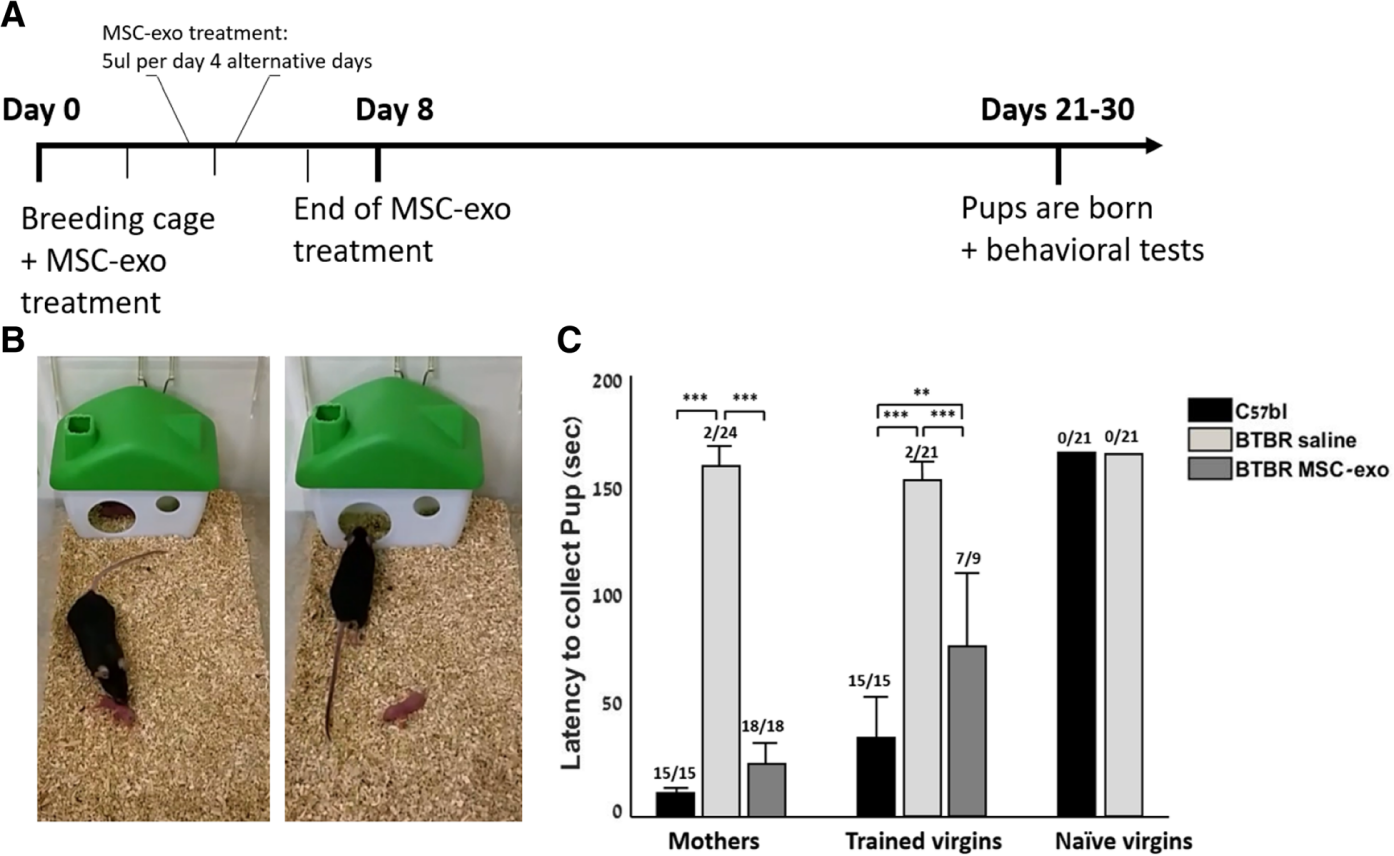
MSC-exo improves maternal pup retrieval and learning of maternal pup retrieval behaviors. **a** Experiment’s timeline. **b** Visualization of normal pup retrieval behavior vs. autistic-like behavior. **c** C57BL and BTBR mice groups were treated with saline or MSC-exo were tested for pup retrieval. Data is presented as mean + SEM. **p < 0.01, ***p < 0.001

We have performed pup-retrieval tests for MSC-exo- or saline-treated mothers, naïve virgins, and experienced virgins [19, 20]. The C57BL saline mothers retrieved all pups (15/15), and their total average time for retrieval was 10.4 s ± 1.08 s. In contrast, only one of eight BTBR saline mothers retrieved two pups (in 156 s), without bringing them back to the nest. Altogether, only 2/24 pups were retrieved. After treatment, BTBR MSC-exo mothers retrieved all pups (18/18) at a mean time of 25.24 s ± 5.7 s (ANOVA1 on time of retrieval, *F*_2,48_ = 332.5, *p* < 0.0001). C57BL saline trained virgins retrieved all pups (15/15) with a total average time of 38.3 s ± 3.3 s. BTBR saline trained virgins mostly did not retrieve the pups besides for one female that had retrieved two pups with a mean time of 152 s. Altogether, 2/21 pups were retrieved by BTBR saline trained virgins. BTBR MSC-exo trained virgins retrieved 7/9 pups at a total average time of 83.3 ± 16.02 s (ANOVA1 on time of retrieval, *F*_2,42_ = 29.47, *p* < 0.0001). Neither the C57BL naïve virgins (0/21) nor the BTBR naïve virgins had retrieved the pups (0/21) (Fig. 4c, Additional files 3 and 4). We had assumed that the MSC-exo naïve virgins would also not retrieve the pups.

### Visualization of MSC and exosomes after intranasal delivery

**Fig. 5:**
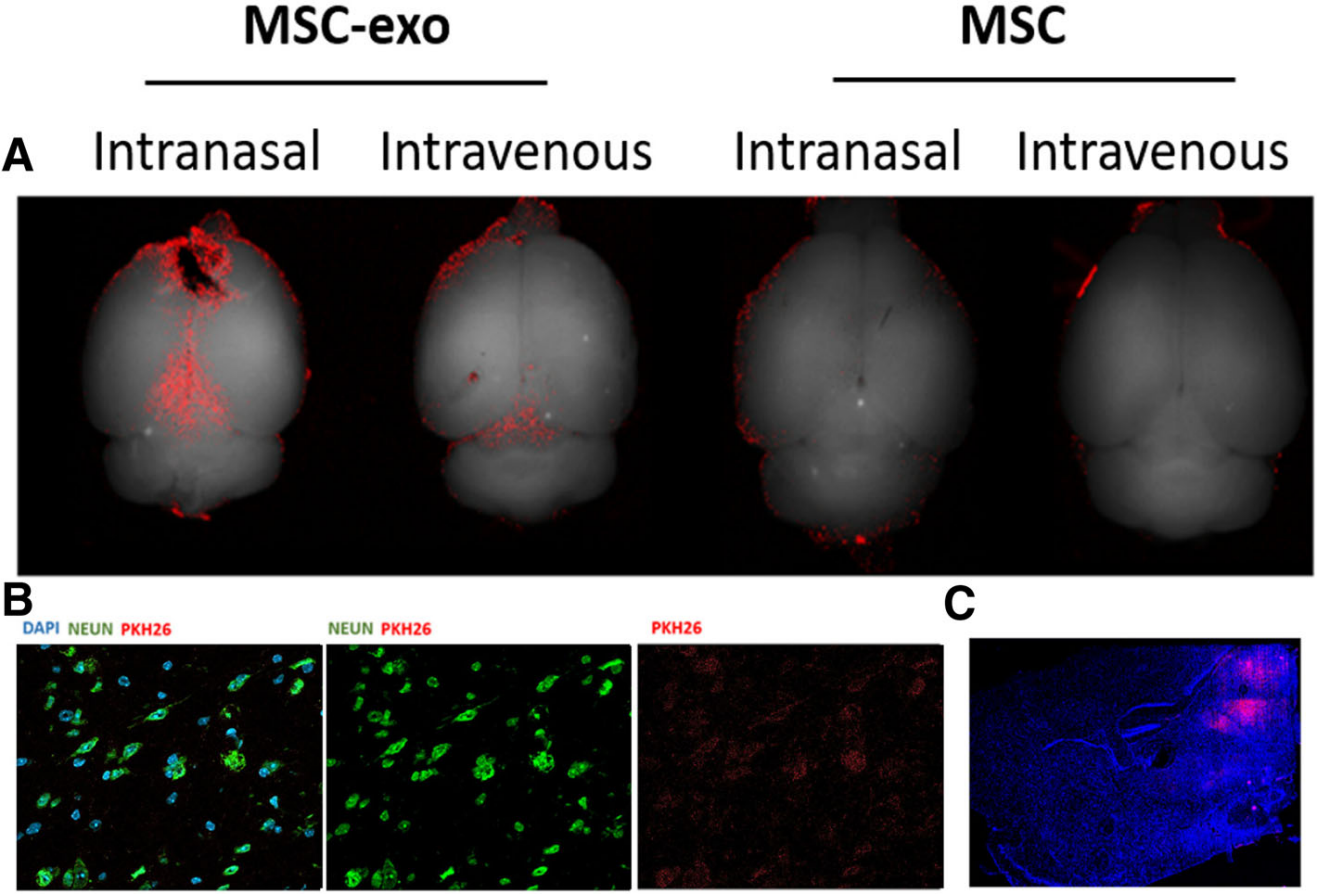
Comparison between MSC-exo and MSC labeled with PKH26 visualization in the brain. **a** From left to right: MSC-exo intranasal, MSC-exo intravenous, MSC intranasal, and MSC intravenous. MSC-exo cross the blood brain barrier, both intranasally and intravenously, while MSC much less. **b** Immunostaining of neurons, DAPI and PKH26 labeled MSC-exo after intranasal administration. **c** Sagittal section of the BTBR MSC-exo brain after intranasal administration of PKH26 labeled MSC-exo (top) and Alan brain atlas (bottom)

MAESTRO whole brain imaging was used to visualize PKH26-labeled MSC and MSC-exo. After intranasal and intravenous administration, MSC-exo can be visualized fluorescently while MSC cannot be detected (Fig. 5a). Immunostaining indicated that MSC-exo penetrate the brain parenchyma and are found in the cells of the tissue (Fig. 5b, c).

### MSC-exo migration and efficacy are dependent on their membrane proteins

**Fig. 6:**
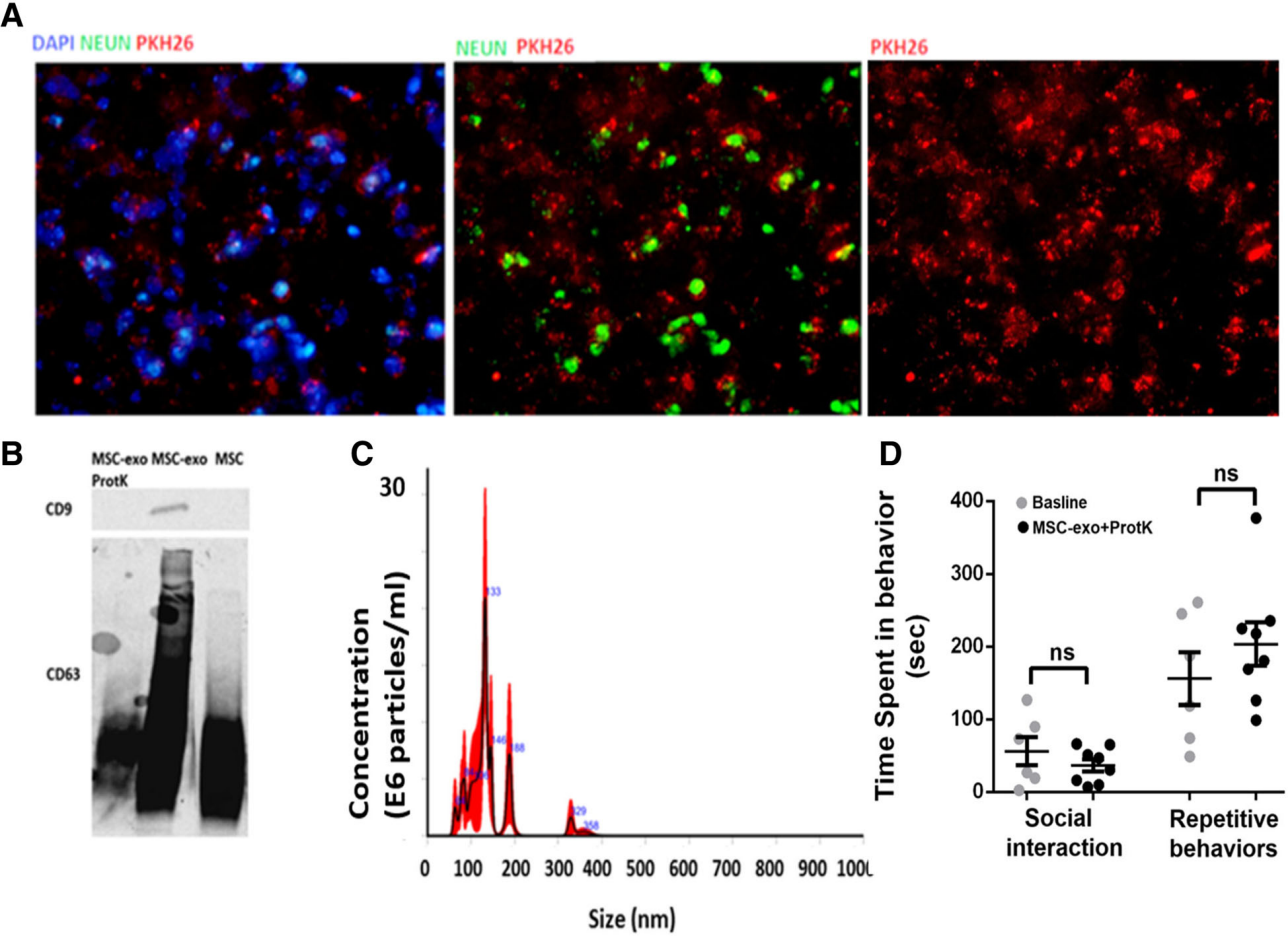
MSC-exo-protK was not found inside the cells and the mice did not present behavioral differences. **a** MSC-exo-protK were mostly located outside of the cells, unlike MSC-exo (Fig. 5b). **b** Western blot of MSC-exo-protK show deletion of CD9 and reduce CD63 compared to MSC-exo. **c** NanoSight analysis of MSC-exo-protK showed no significant change in number and size of MSC-exo-protK compared to MSC-exo (Fig. 1). **d** BTBR mice treated with MSC-exo-protK did not present behavioral improvement compared to saline treated in social and repetitive behaviors

The ability of MSC-exo to cross the blood-brain barrier and to be uptake by cells in the brain is critical for the therapeutic effect. To block the MSC-exo from being uptake by the cells in the brain and to use them as control, MSC-exo were treated with protease-k (ProtK) in order to remove the membrane proteins. Immunostainings of the tissue indicated that the MSC-exo-protk delivered intranasally have not been uptake into the cells at the same efficiency as MSC-exo (Fig. 6a as compared to Fig. 5b). To demonstrate the effectiveness of ProtK in degrading the proteins on the exosomes’ membrane, we used Western blot analysis to CD9 and CD63 since they are known to be overexpressing on the membrane of the exosomes and are commonly used for exosomes detection and characterizations [42]. The Western blot showed deletion of the membrane proteins CD9 and a reduction of CD63 (Fig. 6b). NanoSight analysis showed no significant change in the number and size of MSC-exo-protK compared to MSC-exo (Fig. 6c). BTBR mice that were treated with MSC-exo-protK did not demonstrate behavioral differences in social interaction and repetitive behaviors (Fig. 6d).

## Discussion

We have previously found that surgical transplantation of MSC to the brain ameliorates the autistic-like behaviors of BTBR mice [4]. Furthermore, we have shown that this effect could last for 6 months post a single treatment, even though MSC does not survive for an extended period in the brain [5]. We have also demonstrated that MSC-exo efficiently cross the blood-brain barrier after intranasal administration compares to intravenous injection, and can be visualized in vivo when they are loaded with gold nanoparticles [17]. In the current experiment, BTBR mice were treated via intranasal administration of MSC-exo followed by behavioral tests in all the ASD-like phenotypes presented by this model. Remarkably, the BTBR MSC-exo group presented significant improvements in all the tested ASD-like phenotypes.

In social interaction, the BTBR MSC-exo group spent a significantly longer time engaging in interaction with the stranger male compared to the saline-treated mice and their own basal behaviors. BTBR MSC-exo had also presented a significant decrease in repetitive behaviors of self-grooming and digging compared to their own basal behavior and to the BTBR saline group. Their scores had become closer to the C57BL saline group.

Deficits in social communication are a core symptom of ASD in children [1]. It has been reported that BTBR mice present unusual repertoire of male to female ultrasonic vocalizations compared to normal C57BL mice [21]. We reported that BTBR MSC-exo mice had significant improvement in the number of syllables compared to the control BTBR saline group. Their number of syllables was closer to the C57BL group. To examine other features of ultrasonic vocalizations, such as complexity and classification of syllables, we have used an advanced classification algorithm. This classification revealed significant improvement in the complexity of syllables of BTBR MSC-exo mice, making their ultrasonic vocalizations repertoire closer to the C57BL saline group. Importantly, the improvement in male-to-female ultrasonic vocalizations did not seem to be caused by higher sexual arousal as there was no significant difference between the duration of interaction initiated by the males toward the females in any of the groups. This finding was also observed in our previous studies after MSC transplantation to BTBR mice [5]. Considering the growing recognition that some adolescents and young adults with ASD may exhibit inappropriate sexual behaviors [22, 23], the fact that MSC-exo treatment also does not enhance sexual arousal is an advantage. As compared, the C57BL MSC-exo group did not present behavioral changes in their ultrasonic vocalizations and social interactions toward both males and females. This finding may suggest that MSC-exo administration leads to beneficial effects and not to non-specific hyperactivity or behavioral side effects.

Maternal behavior of BTBR mice has yet to be studied extensively. Here, we observed significant differences between BTBR and C57BL for pup retrieval. C57BL mice exhibit “normal” behavior, and they created a nest for their pups. If the pup was separated, they had quickly retrieved it [18, 19]. In contrast, BTBR created a nest but reacted slowly to the pup separation and nearly always had not brought the pup within 3 min. Interestingly, BTBR MSC-exo mothers demonstrated high scores in the pup retrieval test, with results comparable to C57BL saline mothers. Furthermore, a naïve C57BL virgin who spent a few days with a mother can learn this “experienced” behavior. BTBR saline females did not manage to learn from other mothers (C57BL mothers) while BTBR MSC-exo had presented learning abilities. Maternal behavior, as well as social interaction, acquires high-level synchronization of sensory input and behavioral output. We suggest MSC-exo may play a role in the mechanisms of sensory integration, making its effect influential in the realms of the social domain symptoms. Sensory integration and coordination deficits have been suggested to be one of the underlying mechanism of the ASD patients [24–26].

Stem cell therapy has been previously used on ASD children with long-term beneficial effects [27]. Bone marrow MSC has been proven to be safe to use in several clinical trials [28–30]. Mechanistically, bone marrow MSC was found efficient in promoting tissue regeneration, immunomodulation, and inflammatory reduction [31–33]. We reported that the transplantation of MSC to the lateral ventricles of BTBR mice leads to increased neurogenesis and BDNF in the hippocampus [4]. Although it is clear that MSC have beneficial properties that can be used safely for clinical purposes, recent evidence shows that the therapeutic effect of MSC is largely mediated via the secretion of exosomes that contain important molecular information [34, 35]. Our findings support this concept, and our study demonstrates that a remarkable behavioral effect, on all ASD-like phenotypes of BTBR mice, can be achieved by simply using MSC-exo rather than MSC. We are aware that small number of mice per group is a limiting factor of our results, yet the behavioral difference post MSC-exo treatment is remarkable. Furthermore, in social interaction and repetitive behaviors, each mouse was compared to its own basal behavior and also to saline-treated group post the treatment, this experimental design was chosen to increase the confidence on the results. Mentionable, for the comparison between MSC-exo and NSC-exo, we used another MSC-exo-treated mice; therefore, the USV and social and repetitive behaviors post MSC-exo treatment were tested twice independently.

The contents of MSC-exo have been characterized using proteomics and RNA sequencing. Importantly, the capacity of the exosomes seems to be selectively packaged. Therefore, some of the proteins and miRNA that are over expressed in the cells are packaged in exosomes while others have over-representation [14, 36]. While exosomes contain numerous variations of RNA, their miRNA repertoire has been spotlighted as a major candidate for their effect in the host cell and the tissue. For example, miRNA-143 was found to be related to the immunomodulatory effect of MSC, and miRNA-10b was found related to their migration abilities [37, 38]. In addition, multiple miRNAs highly represented in MSC-exo (miR-191, miR-222, miR-21) regulate cell cycle progression and proliferation and modulate angiogenesis (miR-222, miR-21) [39–41]. Yet, we are aware that MSC-exo are complex vesicles containing hundreds of proteins and RNA molecules; therefore, we cannot pinpoint on the specific factors that led to the behavioral difference and future study may be needed to uncover the mechanism of action that led MSC-exo-treated mice to behavioral amelioration. Moreover, since BTBR mice are multifactorial model of autism, it may be worth to test MSC-exo effect of other models, including genetically modified mice such as shank3 mutation as well.

Altogether, our findings suggest that MSC-exo may be tested as a safe noninvasive treatment to ameliorate behavioral symptoms of patients with ASD.

## Materials and methods

### Mesenchymal stem cells preparation

Human MSC were purchased from Lonza (cat:PT-2501, Basel, Switzerland). Cells were cultured and expanded as previously described [42]. Before exosome collection, the cells were cultured in exosome-free platelets for 3 days and this medium was then collected.

### Exosomes purification protocol

The exosomes were purified by taking the culture fluid and centrifuging it for 10 min at 300*g*. The supernatant was recovered and centrifuged for 10 min at 2000*g*. Once again, the supernatant was recovered and centrifuged for 30 min at 10,000*g*. The supernatant was filtrated through a 0.22-μm filter and centrifuged for 70 min at 100,000*g*. The pellet containing the exosomes and proteins was washed in PBS and then centrifuged for 70 min at 100,000*g*. The pellet containing the purified exosomes was re-suspended in 200 μm of sterilized PBS. Each centrifugation occurred at 4 °C [43].

### Neuronal stem cells preparation

Human neuronal stem cell lines (CTX0E03) were purchased from ReNeuron, UK. Cells were cultured and expanded according to the company’s protocol. Cell lines were routinely cultured at 37 °C in tissue culture flasks freshly coated with mouse laminin (20 μg/ml in DMEM:F12). Growth medium supplemented with 4-OHT was changed three times per week. When 70–90% confluent, cells were passaged using trypsin (0.25%) for 5–10 min at 37 °C, followed by treatment with soybean trypsin inhibitor (0.25 mg/ml). Cells were spun down (800×*g* for 5 min at room temperature) and re-suspended at an appropriate density in full growth medium supplemented with 100 nM 4-OHT. The NSC-exo were purified at the same protocol as MSC-exo, and a number of particles were matched per mouse using NANOsight analysis (Merkel technologies LTD, Israel).

### Exosomes characterization

NanoSight technology (Merkel technologies LTD, Israel) was used to characterize the size and concentration of the exosomes (3.81 × 10^8^ particles/μL). Lysates of MSC-exo were subject for Western blot using SDS polyacrylamide gel. Proteins were transferred to Immobilon®-P membranes (Millipore, Amsterdam, The Netherlands), incubated in 5% milk for 1 h, and probed overnight at 4 °C with CD9 antibody (ABCAM), CD63 (ABCAM), and Calnexin (ABCAM). After three washes in TBS-Tween 20, the membranes were incubated with the secondary antibody for 1 h and re-washed.

### Exosomes labeling

Exosomes were labeled with PKH26 (Sigma-Aldrich) [44, 45]. PKH26 (2 μL) in 500 μL diluent was then added to 50 μL exosomes in PBS for 5 min of incubation. Exosomes were suspended in 70 ml PBS and were centrifuged for 90 min at 100,000*g* at 4 °C. The pellet was suspended in 200 μL of PBS.

### Ex vivo imaging

Adult BTBR male mice (6–7 weeks) were given 5 μL of labeled exosomes or labeled MSC via intranasal administration (*N* = 2 per condition). Another adult BTBR male mice (6–7 weeks) were given 100 μL of intravenously with labeled exosomes or labeled MSC. (The tail vain was warmed using water and exosomes were directly injected to the vain, no anesthetics used, *N* = 2 per condition.) The number of exosomes per mouse was equal between the intranasal/intravenous administrations (19.05 × 10^8^ particles) as well as the number of MSC. For 24-h post administration, mice were perfused and fixated with PBS and 4% paraformaldehyde (PFA). The brains were incubated in PFA for 24 h followed by 30% sucrose for 48 h and stored at 4 °C. Whole brain fluorescence imaging was taken with Maestro CRi, excitation filter 523, and emission filter 560. For immunostaining analysis, the brains were frozen in chilled 2-methylbutane (Sigma-Aldrich), stored at 4 °C, and subsequently sectioned into slices measuring 10 lm. Slides were incubated with blocking solution (5% goat/donkey serum, 1% BSA, 0.5% Triton X-100 in PBS) for 1 h. Thereafter, slides were incubated overnight at 4 °C with primary antibody in blocking solution (mouse anti-NueN, 1:500, Abcam) and secondary antibody in blocking solution (goat anti-mouse Alexa 488, 1:500, Molecular Probes, Invitrogen) for 1–2 h at room temperature. Next, nuclei were counterstained with DAPI (1:500; Sigma-Aldrich). Sections were ultimately mounted with fluorescent mounting solution (Fluoromount-G, Southern Biotech), covered with a cover slide, and sealed with nail polish.

### Proteinase-k treatment to MSC-exo

Two hundred microliters of PKH26 labeled exosomes were incubated with 7 μL proteinase-K (Roche Diagnostica GmbH, Germany) and 750 μL BPS in 55 °C for 10 min [45]. Proteinase-K inhibitor (7 μL) (phenylmethanesulfonyl fluoride, Sigma) was added to the solution and was suspended for 2 h in 70 ml BPS for ultracentrifugation. Finally, the pellet (MSC-exo-protk) was re-suspended in 200ul PBS. For behavioral treatment, non-labeled MSC-exo-protk were used. Proteinase-k-treated MSC-exo were characterized with NANO-sight and Western blot.

### Animals

Mice were placed under a 12-h light/12-h dark condition and grown in individual ventilated cages with access to food and water ad libitum. All experimental protocols were approved by the Tel Aviv University Committee of Animal Use for Research and Education. All methods were performed in accordance with relevant guidelines and regulations. BTBR mice were bred from adult pairs originally purchased from The Jackson Laboratory (Bar Harbor, ME). At 4 weeks of age, the first cohort of littermate male mice was randomly assigned for basal behavioral experiments followed by intranasal administration of saline (BTBR saline, *N* = 7) or MSC-exo (BTBR MSC-exo, *N* = 7). For the pup retrieval experiment, the first cohort of littermate female mice was randomly assigned at 5 weeks of age to saline or to MSC-exo. At the end of the treatment, random groups of females were placed at breeding colonies with BTBR male mice (1 male 2 females, BTBR mothers). At days 1–2 post whelping, random groups of virgin females were placed with the mothers for 4 days to gain experience (BTBR experienced virgins). The rest of the mice were left at their home cage (BTBR naïve virgins) (Additional file 5: Table S1 for number of females in each group).

C57BL mice were bred from adult pairs originally purchased from The Jackson Laboratory (Bar Harbor, ME). At 4 weeks of age, the first cohort of littermate male mice was randomly assigned for basal behavioral experiments followed by intranasal administration of saline (C57BL saline, *N* = 7) or MSC-exo (C57BL MSC-exo). For the pup retrieval experiment, the first cohort of littermate female mice was randomly assigned to saline at 5 weeks of age. At the end of the treatment, random groups of females were placed at breeding colonies with C57BL male mice (1 male 2 females, C57BL mothers). On days 1–2 post-whelping, random groups of virgin females were placed with the mothers for 4 days to gain experience (C57BL experienced virgins). The rest of the mice remained at their home cage (C57BL naïve virgins) (Additional file 5: Table S1 for number of females in each group).

For comparison between NSC-exo and MSC-exo, another group of mice was randomly assigned. They were raised as previously described and treated with intranasal administration of MSC-exo (*N* = 7), NSC-exo (*N* = 5), or saline (*N* = 7). For comparison between saline and MSC-exo+protK in behavioral experiments, another group of mice was randomly assigned. They were raised as previously described and treated with intranasal administration of MSC-exo+protK (*N* = 8) or saline (*N* = 6). Complete summary of all the mice in all the experiments is found in Table S2.

Mice were treated with intranasal MSC-exo or saline for 12 days, 5 μL a day, and every other day (total of 30 μL per mouse). Administration was done using a gentle pipette of 2.5 μL per nostril with no anesthetics.

### Behavioral tests

#### Reciprocal dyadic social interaction test

The reciprocal dyadic social interaction test [46] was conducted using a 5-week-old male C57BL/6 stranger mouse as the social stimulus. The stranger mouse was placed in a 40 × 40 × 20 cm cage with the test mouse. Prior to the test, both mice were isolated for 1 h. In addition, both mice were recorded for 20 min, with the last 10 min quantified by an observer blind to treatment. Cowlog V3 software was used to score the social contact initiated by the test mouse (Helsinki University, Helsinki, Finland). Scoring was determined by the duration that the mice had engaged the stranger mouse in social behaviors. The social behaviors that were quantified included nose to nose sniffing (i.e., approach to the front of the stranger), nose to genital sniffing (i.e., approach to the back of the stranger), and attacking (i.e., test mouse initiates a fight with the stranger mouse). Active avoidance (i.e., test mouse deliberately avoids interaction when the stranger mouse initiates it) was considered an antisocial behavior. During social interactions, the time spent in repetitive behaviors (i.e., self-grooming and digging) was also observed and quantified.

#### Repetitive behaviors not during social interaction

Mice were placed alone in an arena with dimensions 40 × 40 × 20 cm for 20 min. The last 10 min was quantified for grooming and digging. While observing the grooming behavior, the mice were placed in a clean cage absent from wood-chips in order to prevent digging. Also, self-grooming was not measured while observing the digging behavior.

#### Ultrasonic vocalizations

Both BTBR and C57BL males met C57BL/6 females. Each male was placed in a separate cage for 1 h, and a female was then placed in the cage. Ultrasonic vocalizations were recorded for the first five minutes of encounter to prevent extremely high sexual arousal and mating behaviors. The encounters were filmed for male-female interaction analysis. In the study, all males and females were sexually naïve. Females were in the same cage in order to synchronize their estrus cycle and had met the males on the same day. Vocalizations were recorded with Avisoft-RECORDER v. 4.2.21 recording program. The settings included a sampling rate of 250 kHz and a format of 16 bit. For spectrogram generation, recordings were transferred to Avisoft-SASLab Pro Version 5.2.07 and a fast Fourier transformation (FFT) was conducted. Spectrograms were generated with an FFT length of 256 points and a time window overlap of 50% (100% Frame, FlatTop window). The number of syllables was quantified automatically by Avisoft-SASLab, and syllable classification was done by MATLAB using a classification algorithm [47] (Additional file 5: Supplementary Materials and Methods).

#### Pup retrieval

C57BL and BTBR females were tested for their latency to retrieve pups and bring them back to the nest. Each one was tested with three pups, and the duration of each trial was 180 s. In addition, the number of retrieved pups and time required for each pup retrieval was measured. During the test, all females were taken out of the home cage and were placed back one at a time for a 5-min acclimation with the pups prior to testing. Each of the pups was between 1 and 3 days old [18, 19]. The tests were filmed with a SAMSUNG 11mp camera.

## Additional files

Additional file 1: BTBR male control male to male social interaction. (AVI 5543 kb)

Additional file 2: BTBR male MSC-exo male to male social interaction. (mov 6092 kb)

Additional file 3: BTBR mother control pup retrieval. (AVI 5543 kb)

Additional file 4: BTBR mother MSC-exo pup retrieval. (AVI 6092 kb)

Additional file 5: **Figure S1.** MSC-exo decreases repetitive behavior of self-grooming. Intra-subject comparison shows BTBR MSC-exo group spent significantly less time in repetitive behaviors, while BTBR saline and C57BL saline showed no difference between basal and post treatment behavior (paired *T* test). Inter-group comparison shows BTBR MSC-exo is significantly different than BTBR saline group in time spent in repetitive behaviors. (ANOVA1, Bonferroni). Data is presented as mean + SEM. **p < 0.05. **Figure S2.** MSC-exo had no significant effect on C57BL behavior. A. C57BL mice were tested for baseline behavior (baseline) and after MSC-exo intranasal administration (post-treatment) in the tests of social, antisocial interaction, and repetitive behaviors. No significant differences were found in any of the behaviors (paired *T* test, *p* > 0.05). B. C57BL MSC-exo mice presented no difference in their number of USV compared to saline-treated group (unpaired *T* test, p > 0.05). Data is presented as mean + SEM. **Figure S3.** MSC-exo but not NSC-exo significantly ameliorates male to male social interaction, repetitive behaviors, and male to female ultrasonic vocalizations of BTBR mice. A. male to male social interaction. B. repetitive behaviors. C. male to female ultrasonic vocalizations (ANOVA 1, Bonfferoni *p < 0.05, **p < 0.01, ***p < 0.001). Data is presented as mean + SEM. **Figure S4.** Full Western blot gels: A. Calnexin as negative marker for MSC-exo B. CD9 as positive marker of MSC-exo. C. CD63 as positive marker of MSC-exo and for reduction after protK treatment. D. CD9 as positive marker of MSC-exo and for reduction after ProtK treatment. **Table S1.** Number of females tested in maternal behavioral experiment. **Table S2.** Number of mice at each group in the behavioral experiments. (DOCX 1010 kb)

## Abbreviations

ASD: Autism spectrum disorders
BTBR: BTBR T+tf/J
MSC: Mesenchymal stem cells
MSC-exo: Exosomes secreted from MSC
