# Supplementary material for: Intranasal administration of exosomes derived from mesenchymal stem cells ameliorates autistic-like behaviors of BTBR mice
Source: Mol Autism. 2018 Nov 21;9:57. doi: 10.1186/s13229-018-0240-6 (PMC6249852; doi:10.1186/s13229-018-0240-6)
Supplement: Supplementary file 5 — Figure S1. MSC-exo decreases repetitive behavior of self-grooming. Intra-subject comparison shows BTBR MSC-exo group spent significantly less time in repetitive behaviors, while BTBR saline and C57BL saline showed no difference between basal and post treatment behavior (paired T test). Inter-group comparison shows BTBR MSC-exo is significantly different than BTBR saline group in time spent in repetitive behaviors. (ANOVA1, Bonferroni). Data is presented as mean + SEM. **p < 0.05. Figure S2. MSC-exo had no significant effect on C57BL behavior. A. C57BL mice were tested for baseline behavior (baseline) and after MSC-exo intranasal administration (post-treatment) in the tests of social, antisocial interaction, and repetitive behaviors. No significant differences were found in any of the behaviors (paired T test, p > 0.05). B. C57BL MSC-exo mice presented no difference in their number of USV compared to saline-treated group (unpaired T test, p > 0.05). Data is presented as mean + SEM. Figure S3. MSC-exo but not NSC-exo significantly ameliorates male to male social interaction, repetitive behaviors, and male to female ultrasonic vocalizations of BTBR mice. A. male to male social interaction. B. repetitive behaviors. C. male to female ultrasonic vocalizations (ANOVA 1, Bonfferoni *p < 0.05, **p < 0.01, ***p < 0.001). Data is presented as mean + SEM. Figure S4. Full Western blot gels: A. Calnexin as negative marker for MSC-exo B. CD9 as positive marker of MSC-exo. C. CD63 as positive marker of MSC-exo and for reduction after protK treatment. D. CD9 as positive marker of MSC-exo and for reduction after ProtK treatment. Table S1. Number of females tested in maternal behavioral experiment. Table S2. Number of mice at each group in the behavioral experiments. (DOCX 1010 kb) [file 13229_2018_240_MOESM5_ESM.docx]

Intranasal administration of exosomes derived from mesenchymal stem cells ameliorates autistic-like behaviors of BTBR mice

**Authors:** Nisim Perets^1^, Stav Hertz^2^, Michael London^2^, Daniel Offen^1,3^

**Affiliations:**

^1^Sagol School of neuroscience, Tel Aviv University, Israel.

^2^Edmond and Lily Safra center for brain sciences, Hebrew University, Jerusalem, Israel.

^3^Sacklar school of medicine, department of human genetics and biochemistry Tel Aviv University, Israel.

**Supplementary Materials**

Materials and Methods

Syllable classification algorithm:

1. Detect and attenuate noisy frequencies
   - Some recordings have a noise that appears throughout the file in a single or multiple adjacent frequencies. The first step of the algorithm detects and removes it.
2. Remove low amplitude frequencies in each column
   - The first threshold that is performed is done according to amplitude of the frequencies in each time point. All low amplitudes are set to silence.
3. Clear isolated frequencies
   - Active frequencies that do not have any active frequencies in the area that surrounds them are set to silence.
4. Collect all columns that contain frequencies with positive amplitude
   - Due to the previous steps, only columns that contain frequencies that are part of a syllable remain. We collect all these in order to examine the gaps between them and divide them into syllables and sequences.
5. Separate the columns to syllable and sequences
   - The separation is done according to predetermined gap size thresholds that were calculated based on the statistics of the data.
6. Return the start and end time of each syllable and sequence for further analysis.
   - These values are also store in a large database containing many syllables from many recording sessions.


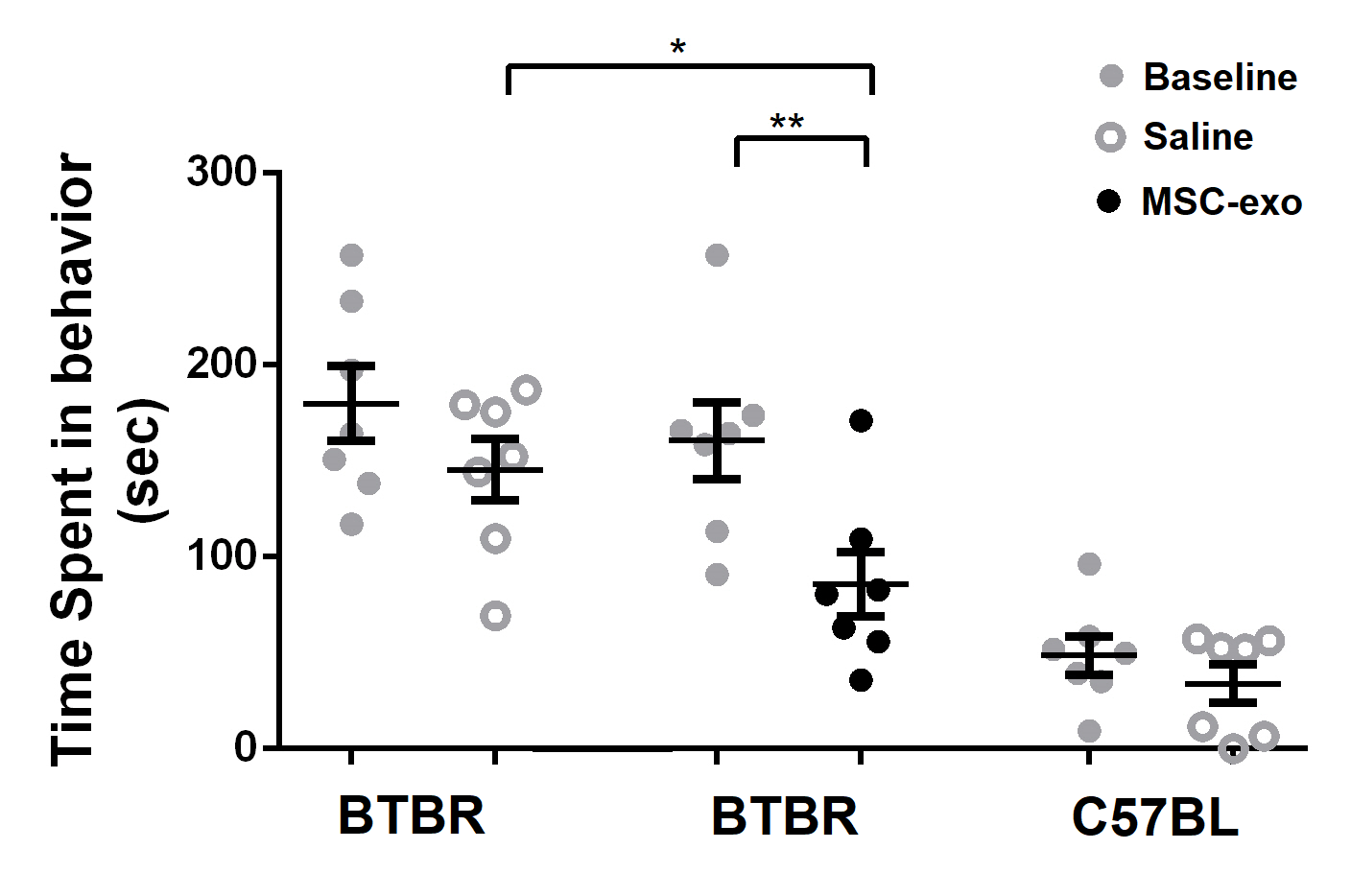


Figure S1. MSC-exo decreases repetitive behavior of self-grooming. Intra-subject comparison shows BTBR MSC-exo group spent significantly less time in repetitive behaviors, while BTBR saline and C57BL saline showed no difference between basal and post treatment behavior (paired T-test). Inter-group comparison shows BTBR MSC-exo is significantly different than BTBR saline group in time spent in repetitive behaviors. (ANOVA1, Bonfferoni). Data is presented as mean+SEM. **p<0.05.


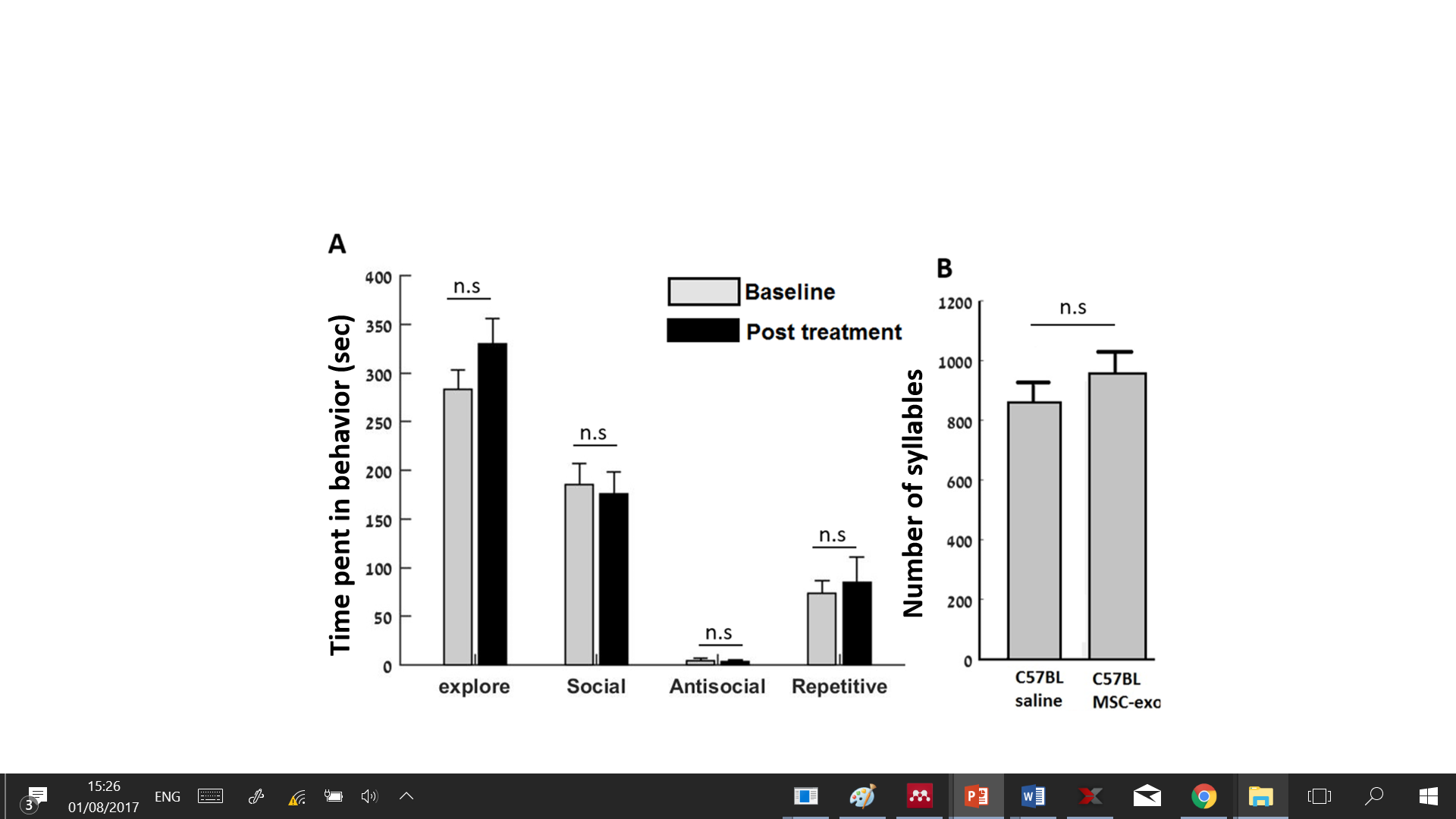


Figure S2. MSC-exo had no significant effect on C57BL behavior. A. C57BL mice were tested for baseline behavior (baseline) and after MSC-exo intranasal administration (post-treatment) in the tests of social,

antisocial interaction, and repetitive behaviors. No significant differences were found in any of the behaviors (paired T-test, p>0.05). B. C57BL MSC-exo mice presented no difference in their number of USV compared to saline treated group (unpaired T-test, p>0.05). Data is presented as mean+SEM.

.

**
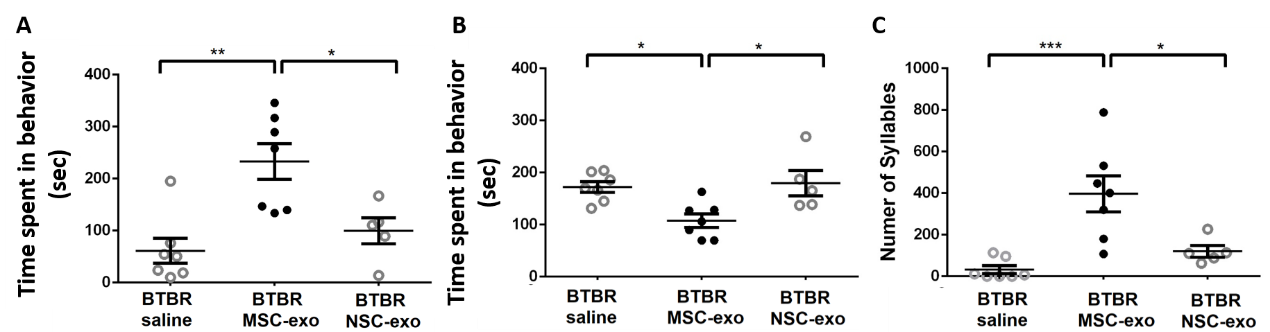
**

Figure S3. MSC-exo but not NSC-exo significantly ameliorates male to male social interaction, repetitive behaviors, and male to female ultrasonic vocalizations of BTBR mice. A. male to male social interaction. B. repetitive behaviors. C. male to female ultrasonic vocalizations (ANOVA 1, Bonfferoni *p<0.05, **p<0.01, ***p<0.001). Data is presented as mean+SEM.

Table S1. Number of females tested in maternal behavioral experiment

| Females tested in behavioral experiments | | | | |
| --- | --- | --- | --- | --- |
|  |  | mothers | Trained virgins | Naïve virgins |
| BTBR | MSC-exo | 6 | 3 | - |
|  | Saline | 8 | 7 | 7 |
| C57BL | Saline | 5 | 5 | 7 |

Table S2. Number of mice at each group in the behavioral experiments.

| Males tested in behavioral experiments | | |
| --- | --- | --- |
| BTBR | MSC-exo | 14 |
|  | Saline | 20 |
|  | MSC-exo+protK | 8 |
|  | NSC-exo | 5 |
| C57BL | Saline | 13 |
|  | MSC-exo | 6 |


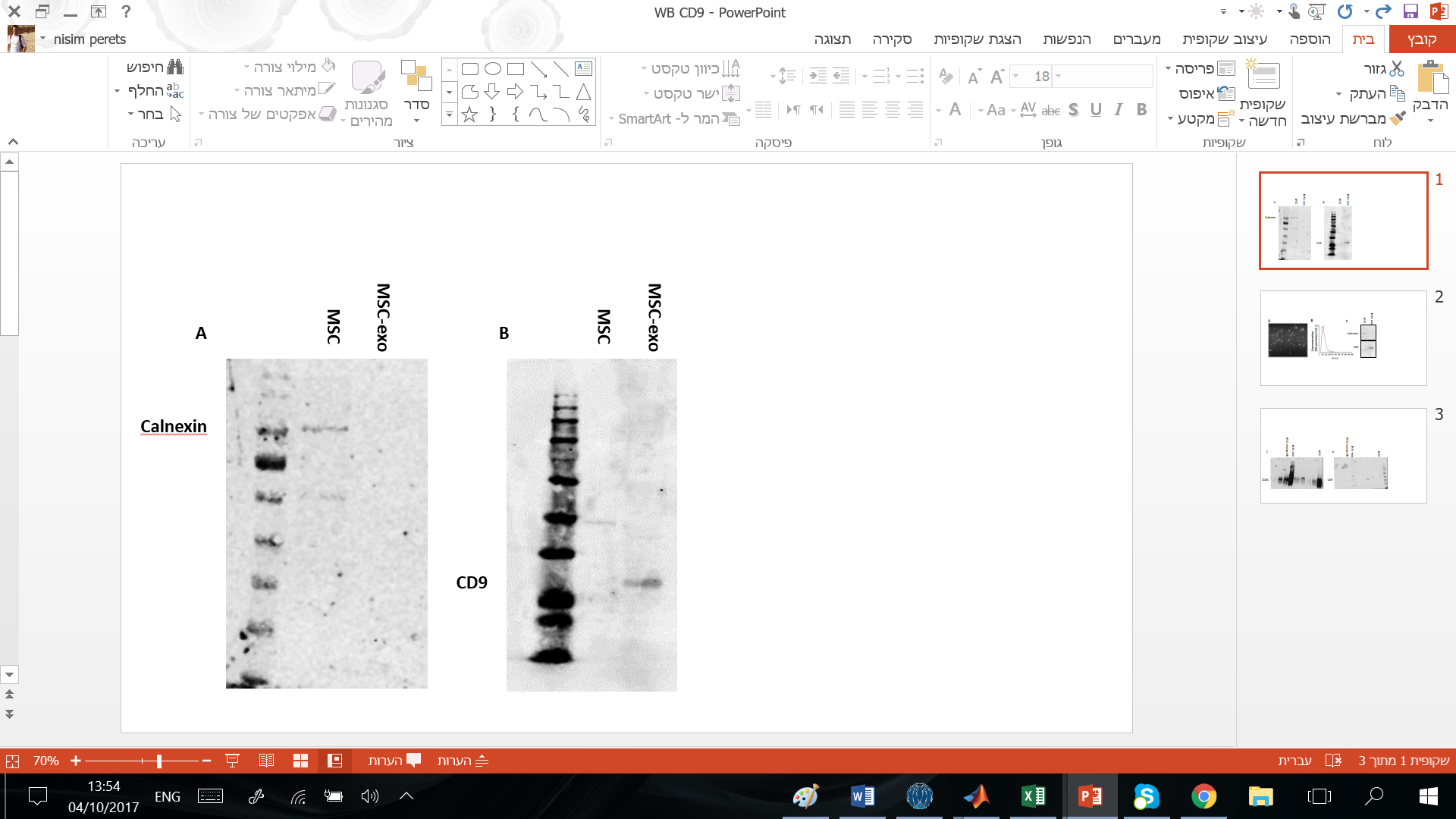


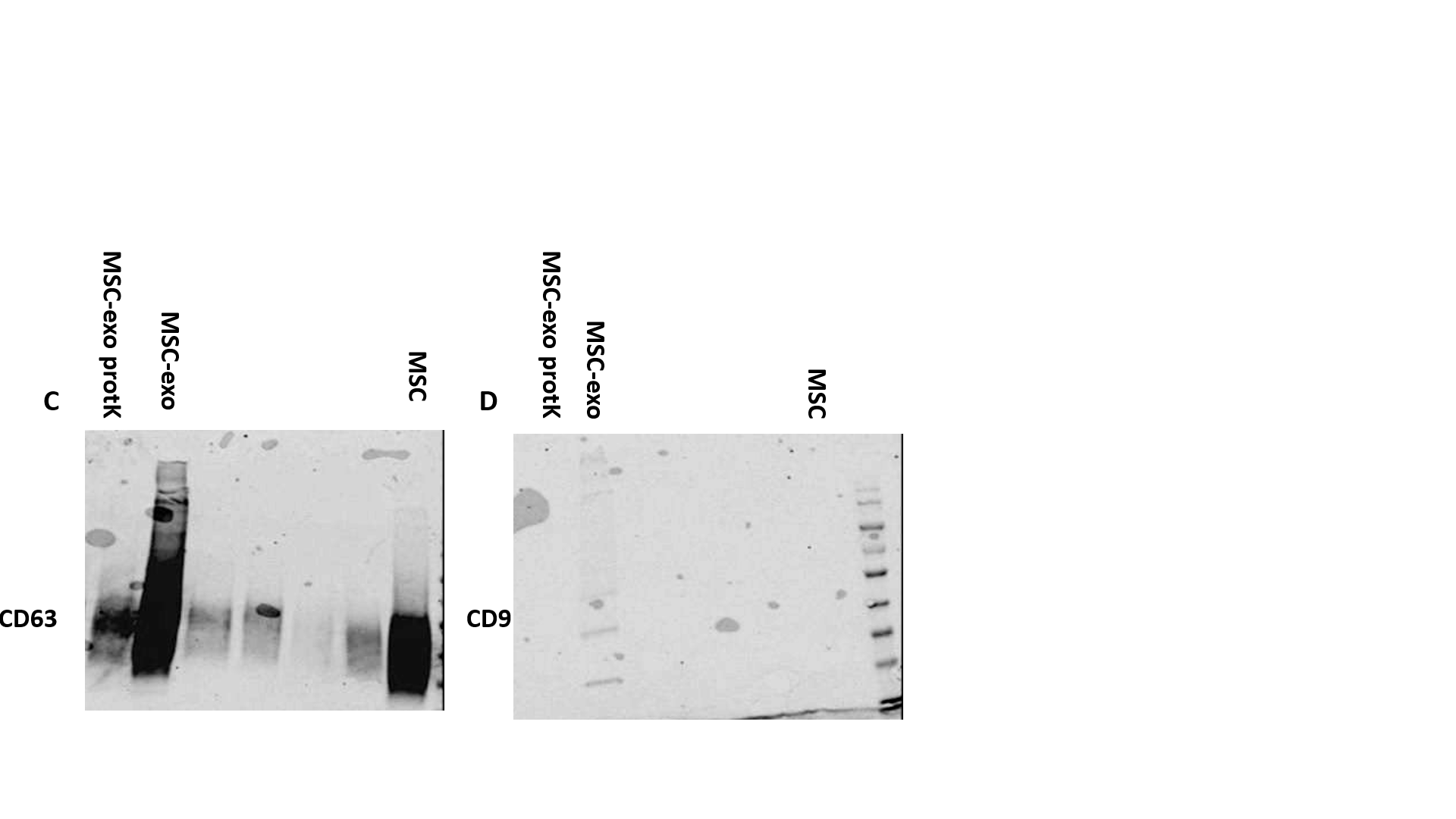


Figure S4. Full Western blot gels: A. Calnexin as negative marker for MSC-exo B. CD9 as positive marker of MSC-exo. C. CD63 as positive marker of MSC-exo and for reduction after protK treatment. D. CD9 as positive marker of MSC-exo and for reduction after ProtK treatment.
